# Research and Experimentation on Acoustic Monitoring Technology for Laser Drilling Penetration

**DOI:** 10.3390/mi16040475

**Published:** 2025-04-16

**Authors:** Bowen Lian, Kewen Pan, Liqun Wang, Liwu Shi, Jianhua Yao, Wei Guo

**Affiliations:** 1 College of Mechanical Engineering, Zhejiang University of Technology, Hangzhou 310023, China; 2 Laser Extreme Manufacturing Research Center, Ningbo Institute of Materials Technology and Engineering, Chinese Academy of Sciences, Ningbo 315201, China

**Keywords:** laser drilling, penetration monitoring, back wall damage, acoustic monitoring

## Abstract

To prevent back wall damage in cavity workpieces during laser drilling, it is crucial to monitor hole penetration status in real time. This study proposes a laser drilling penetration monitoring method based on acoustic principles. First, the acoustic module parameters of the system were simulated and calibrated using COMSOL Multiphysics (version 6.1) software, resulting in an optimal sound source frequency of 35 kHz and an incident angle of 30° for the acoustic waves. Next, a nickel-based alloy laser drilling acoustic monitoring platform was designed and constructed, and the system’s upper computer control software was developed. Subsequent drilling trials were performed on the validated platform. During the experiments, the threshold for hole penetration signals under the specified experimental parameters was determined, enabling the acquisition of acoustic signals and the identification of hole penetration status. Furthermore, a correlation between the intensity of the acoustic signals and the exit aperture of the drilled holes was established. The results demonstrate the feasibility of using acoustic principles to monitor hole penetration status and measure machining aperture, providing both theoretical and experimental foundations for active laser drilling to prevent back wall damage.

## 1. Introduction

In recent years, the rapid development of industries such as aerospace, precision manufacturing, and medical devices has increasingly highlighted the critical role of micro-holes in enhancing product performance and achieving specific functionalities. Laser drilling technology, renowned for its exceptional processing efficiency, superior machining quality, broad material applicability, and non-contact processing advantages, has emerged as a pivotal solution in fields such as micro-hole manufacturing for aeroengine components [1,2,3]. The fundamental principle of this technology involves the emission of a high-energy-density laser beam from a laser source, which is then focused by a lens to create a minute high-temperature zone on the material surface. This causes the material to rapidly reach a molten or vaporized state. Subsequently, the molten material is expelled under the influence of high-pressure gas and vaporized byproducts, forming a micro-hole in the material. After the laser irradiation ceases, the molten material along the hole walls solidifies, ultimately resulting in a micro-hole with the desired shape [4,5,6].

However, when processing workpieces with complex internal structures, such as aeroengine turbine blades, laser machining faces significant challenges. These blades feature hollow interiors, confined spaces, and intricate curved surfaces. Improper operation may lead to excessive machining of the inner cavity walls after the laser beam penetrates the material’s lower surface, resulting in so-called back wall damage. Back wall damage not only reduces the material’s fatigue life but also adversely affects its hardness and tensile strength, thereby severely compromising the overall performance and service life of the turbine blades. Consequently, implementing effective protective measures to prevent back wall damage has become crucial for ensuring machining quality. To address the issue of back wall damage and enhance the quality and efficiency of laser machining, current auxiliary methods for back wall damage prevention in laser drilling primarily fall into two categories: passive protection by adding protective materials and active protection using various sensors to monitor the laser drilling process [3,7,8,9].

The former involves selecting suitable protective materials and filling them into the internal cavities of the workpiece [10]. For instance, Turner [11] utilized fine crystalline materials to fill internal cavities, achieving back wall damage prevention and absorbing internal splashes. Corfe [12] coated the inner cavity surfaces with fine granular polytetrafluoroethylene (PTFE), which was removed by heating and melting after laser machining. Ma. K [13] employed an aqueous suspension composed of acrylamide and acrylic acid particles to fill the cavities, which dispersed and attenuated the laser energy, thereby preventing back wall damage. Wang Bin [14] filled cavities with flowing polyacrylamide (PAM), polyurethane (PUR), and paraffin, finding that flowing PAM provided superior protection compared to other materials. At a flow rate of 3.0 m/s and cavity spacings of 0.5–3 mm, no back wall damage occurred within 50 s. Tian Dongpo [15] filled cavities with corundum and investigated the process of damage-free drilling on the back walls of samples and blades, demonstrating that this approach achieved protection without compromising micro-hole machining quality. However, these existing mitigation approaches necessitate the infusion of diverse protective materials into cavity interiors, inevitably compromising processing efficiency. Furthermore, their protective efficacy remains inconsistent due to dependencies on both laser parameter optimization and the thermophysical quality of shielding substances. These dual constraints fundamentally limit practical implementation viability in high-precision manufacturing scenarios.

The latter approach involves selecting different types of sensors to detect signals such as sound, light, and heat generated during the interaction between the laser and the material, thereby enabling real-time monitoring of the drilling process [16,17]. For example, Aristidis [18] studied the correlation between signals received by acoustic sensors and the geometric characteristics of holes, finding a strong correlation between signal amplitude and hole depth. They validated the feasibility of using acoustic signals acquired during drilling to monitor the process. Yan Qing [19] employed laser-induced breakdown spectroscopy (LIBS) monitoring technology, selecting Cr (I) 521.531 nm as the characteristic emission spectrum. By using changes in spectral signal intensity during drilling to determine penetration status, they verified the feasibility of this method in avoiding back wall damage during hole machining. Aristidis [20] used photodiodes to monitor the output signal intensity at various stages of laser drilling and investigated the relationship between photoelectric signals and hole depth and entrance diameter, concluding a strong correlation between photoelectric signals and hole diameter.

This study adopts an acoustic monitoring method with a signal source, determining the drilling stage by monitoring acoustic signals of known frequency and phase before and after penetration. On one hand, based on the wave equation and transmission characteristics of acoustic waves, since the acoustic impedance of metal is significantly higher than that of air (typically 10,000 times greater), when a planar acoustic wave impinges on a metal surface, nearly all waves are reflected with negligible transmission. This implies that when the workpiece is not penetrated by the laser, almost no signal is detected by the microphone. However, at the instant of laser-induced breakthrough, acoustic waves can pass through the small hole and be captured by the microphone. The entire monitoring process is completed within milliseconds and remains unaffected by material properties.

On the other hand, this monitoring system employs a lock-in amplifier for signal acquisition and processing. The lock-in amplifier’s capability to lock onto and track fixed-frequency phase-referenced signals enables selective detection of the specific reference signal emitted by the loudspeaker, effectively rejecting interference from other noise sources.

Compared to acoustic monitoring methods without a signal source, this approach effectively eliminates interference noise, enhancing monitoring accuracy. While methods using piezoelectric transducers to monitor shock waves can predict drilling depth effectively, they impose stringent experimental conditions, influenced by factors such as the optical and thermal properties of the material, material thickness, and laser wavelength. Otherwise, the relationship between drilling depth and the number of laser pulses cannot be detected. In contrast, the method proposed in this study imposes almost no special requirements on the material’s physical or chemical properties, nor on the choice of laser or laser parameters. Methods relying on monitoring reflected laser brightness or characteristic spectral line intensity exhibit a slow, cumulative change in brightness or spectral lines, lacking a clear distinction before and after penetration. This makes them susceptible to human error, leading to incomplete or excessive penetration. Additionally, they are similarly influenced by the material’s physical or chemical properties. Furthermore, optical monitoring methods using penetration sensing technology involve complex processes, including image input, preprocessing, feature extraction, and analysis, significantly affecting the system’s overall response speed. In comparison, the method proposed in this study exhibits distinct signal differences before and after penetration, with monitored signals directly input into the computer for analysis and calculation, achieving a response time in milliseconds. This enables rapid and accurate determination of whether penetration is complete.

In this study, based on the established acoustic detection platform for laser drilling of nickel-based alloys, the acoustic system was first simulated using COMSOL software. Subsequently, the upper computer control software for the detection system was designed using LabVIEW (Version 2022) programming software to achieve automated monitoring during the laser drilling process. Finally, experiments were conducted to validate the feasibility of using the acoustic detection method for determining hole penetration during the laser drilling process.

## 2. Principle and Simulation of Acoustic Monitoring System for Laser Machining

### 2.1. Principle of the Acoustic Monitoring System for Laser Machining

**Figure 1:**
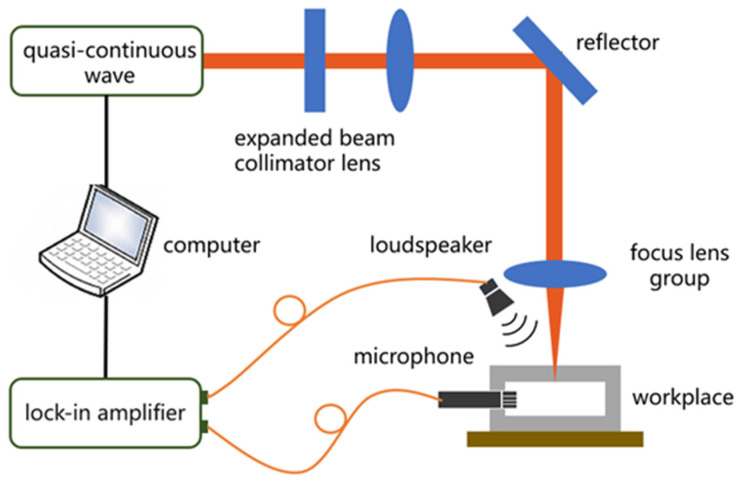
Schematic Diagram of the Monitoring System.

**Figure 2:**
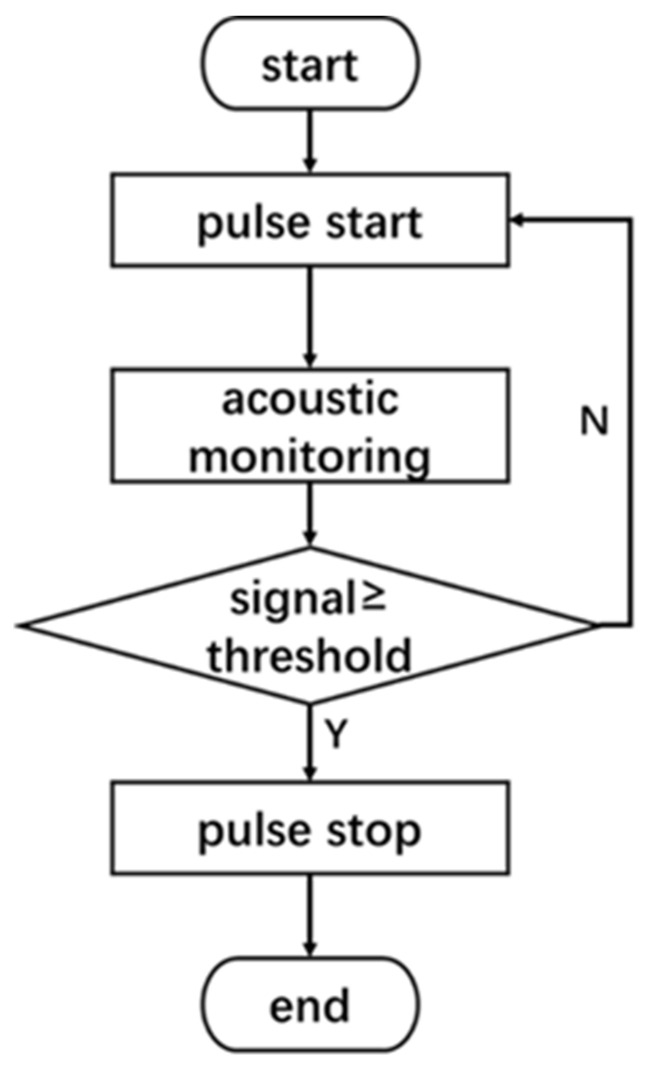
Flowchart of the Monitoring System.

This monitoring system employs an acoustic method with a signal source to monitor the acoustic signals throughout the entire laser drilling process. The collected signal data is then processed and analyzed using virtual instrument software and lock-in amplifier (SR830, Stanford Research Systems, Sunnyvale, CA, USA) to ultimately achieve hole penetration monitoring [21]. Before acquiring the target acoustic signal, the acoustic signal emitter (W10726606, SWAN, Shenzhen, China) must be appropriately positioned above the workpiece to emit a reference signal with known phase, frequency, and amplitude. An acoustic signal acquisition sensor (TYPE4966, Brüel & Kjær, Naerum, Denmark) is placed inside the cavity below the workpiece. A schematic diagram of the monitoring system’s principle is shown in Figure 1. This detection system uses the reference signal emitted by the signal source as the target monitoring signal. According to the principles of acoustic wave propagation, before the laser penetrates the workpiece, the acoustic wave reflects off the metal surface, reducing the longitudinal propagation velocity to zero, and the microphone receives almost no reference signal. When the laser penetrates the workpiece, the reference signal propagates through the air in the hole into the cavity and is received by the microphone. At this point, the acoustic signal is converted into an electrical signal. The subsequent data acquisition system collects, processes, analyzes, and saves the signals acquired by the microphone. Penetration is determined by comparing the target reference signal during the drilling process with a predefined threshold [22,23]. A detailed flowchart of the monitoring system is shown in Figure 2.

### 2.2. Acoustic System Simulation

**Figure 3:**
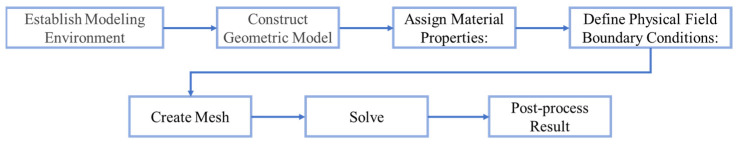
Modeling and Simulation Flowchart.

To determine the parameters such as the signal frequency and incident angle of the sound source used in the experiments, the acoustic system was simulated using COMSOL software. The Figure 3 shows the modeling and simulation flowchart using COMSOL finite element analysis software [24,25].

This study investigates the propagation of pressure waves from the signal source through the small hole in the workpiece to the cavity structure, which constitutes a highly complex three-dimensional acoustic problem involving multiple acoustic cavities and channels separated by thin, rigid walls. To analyze the attenuation of harmonic pressure waves, including sound transmission through small holes, a multi-physics approach combining pressure acoustics and thermos-viscous acoustics was employed. Within the pressure acoustics module, the frequency-domain interface uses a modified Helmholtz equation to solve for the acoustic pressure *p*:

(1) $$\nabla \cdot \left(-\frac{\nabla p}{\rho }\right)-\frac{\omega^{2}p}{c^{2}\rho }=0$$

where:

ρ is the density,
c is the speed of sound,
ω is the angular frequency.

In the thermos-viscous acoustics module, the governing equations are derived from the Navier-Stokes equations:

(2) $$\rho \left(\frac{𝜕T}{𝜕t}+v\cdot \nabla v\right)=-\nabla p+\mu \nabla^{2}v+\rho g$$

where:

μ is the dynamic viscosity,
g is the gravitational acceleration.

**Figure 4:**
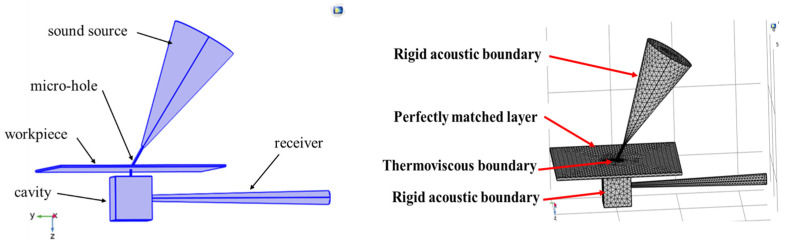
Simulation Model and boundary conditions Diagram.

Next, a geometric model was constructed as shown in the Figure 4, and different types of boundary conditions were defined, including hard sound field boundaries, thermos-viscous boundaries, and perfectly matched layer (PML) boundaries. Finally, the solver was configured, and post-processing of the results was performed.

During post-processing, to define the transmission loss, the incident and outgoing plane waves were assumed to superimpose at the inlet boundary:

(3) $$\left(-\frac{\nabla p}{\rho }\right)\cdot n=\frac{i\omega }{\rho c}p-\frac{2i\omega }{\rho c}p_{0}$$

where:

*p*_0_ represents the applied external pressure,
i is the imaginary unit.

At the outlet boundary, an outgoing plane wave was set:

(4) $$\left(-\frac{\nabla p}{\rho }\right)\cdot n=\frac{i\omega }{\rho c}p$$

The acoustic effects at the signal inlet and outlet were defined as:

(5) $$P_{in}=\int_{𝜕\Omega }^{1}\frac{{p_{0}}^{2}}{2\rho c}dA$$

(6) $$P_{out}=\int_{𝜕\Omega }^{1}\frac{{\left|p_{c}\right|}^{2}}{2\rho c}dA$$

The transmission loss for the entire process was then calculated as:

(7) $$TL=10\log\left(\frac{p_{in}}{p_{out}}\right)$$

This comprehensive approach enables the accurate simulation and analysis of pressure wave propagation, including transmission loss, in complex geometries with small holes and cavities [26,27].

**Figure 5:**
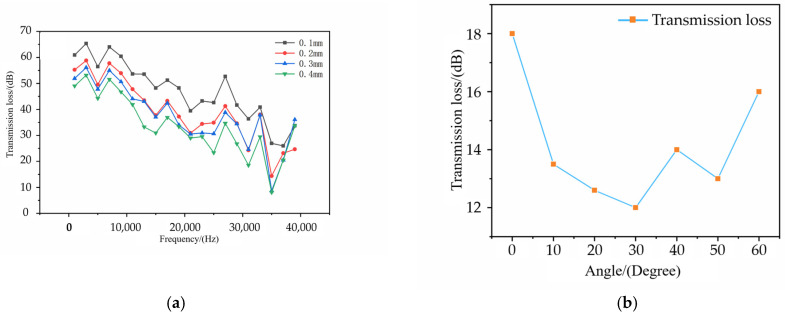
Simulation result diagram: (**a**) Frequency Impact Diagram; (**b**) Incidence Angle Impact Diagram.

The simulation parameters were set with p0 at one standard atmosphere. The analysis examined the effects of different hole radius on transmission loss and output power across a frequency range of 0 Hz to 40 kHz, in increments of 1 kHz. The results are shown in Figure 5.

From Figure 5a, it can be observed that under all parameters, the system’s transmission loss remains below 70 dB. As the frequency of the sound source signal increases, the transmission loss generally decreases, reaching its minimum at 35 kHz. Beyond 35 kHz, the transmission loss slightly increases. This is because higher-frequency acoustic signals exhibit better directivity, with energy being more concentrated and less susceptible to noise interference. Additionally, high-frequency acoustic waves have shorter wavelengths, which can provide higher spatial resolution, facilitating precise measurement and monitoring. However, excessively high frequencies result in wavelengths that are too short, reducing transmission distance and increasing absorption and attenuation during propagation. Moreover, generating and monitoring such high-frequency acoustic waves requires sensors and transducers with higher frequency responses, imposing greater hardware requirements. Therefore, a sound source frequency of 35 kHz was selected for subsequent experiments.

Figure 5b shows the transmission loss corresponding to different angles of acoustic wave incidence at 35 kHz. The transmission loss initially decreases and then increases as the angle grows, reaching its minimum at 30°. Therefore, in the actual experiments, the incident angle of the sound source was set at 30° relative to the laser emission angle to achieve optimal acoustic transmission efficiency.

## 3. Acoustic Monitoring Experiment for Hole Penetration

The principle of sound generation from the interaction between laser and material is mainly that after the laser energy is absorbed by the material, it causes material vibration or air disturbance through ways such as thermal expansion, plasma shock waves, phase transformation, or microscopic energy transfer, thereby forming sound waves. This paper mainly uses sound sensors to monitor known reference signals to determine the laser drilling stage, which is not affected by other noises [28,29].

### 3.1. Experimental Material and Parameters

#### 3.1.1. Experimental Materials

To simulate the laser drilling process of aeroengine turbine blades as closely as possible, this experiment used GH4169 high-temperature nickel-based alloy as the workpiece material. The dimensions of the workpiece were 50 mm × 50 mm × 4 mm, and its chemical composition is detailed in the Appendix A. To avoid measurement errors caused by variations in surface roughness, the test samples were ground, polished, and ultrasonically cleaned before the experiment, ensuring a smooth surface condition [30,31]. The chemical composition of the materials is detailed in the Table 1.

**Figure A1:**
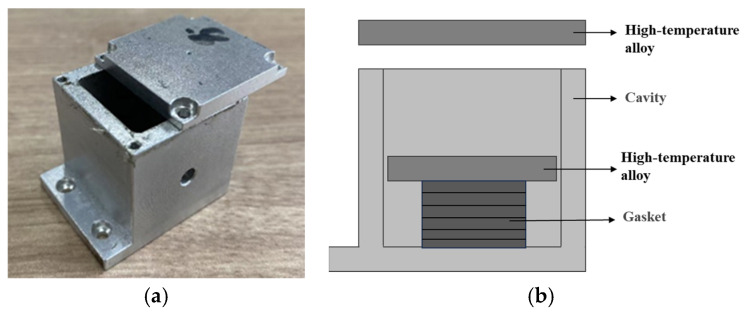
Diagram of cavity structure (**a**) Physical Object Diagram; (**b**) Cross-sectional Diagram.

To replicate the internal cavity structure of real aeroengine turbine blades, a custom aluminum cavity chamber was designed. The actual and cross-sectional views of the chamber are shown in the Appendix A. The top opening of the cavity allows for the replacement of the nickel-based alloy workpiece. A hole was drilled on the left side of the cavity to accommodate the signal receiver for monitoring. A nickel-based alloy plate, identical to the upper workpiece, was placed 3 mm below the upper workpiece to simulate the backwall inside the turbine blade cavity. The actual and cross-sectional views of the chamber are shown in the Figure A1.

#### 3.1.2. Experimental Parameter Settings

**Figure 6:**
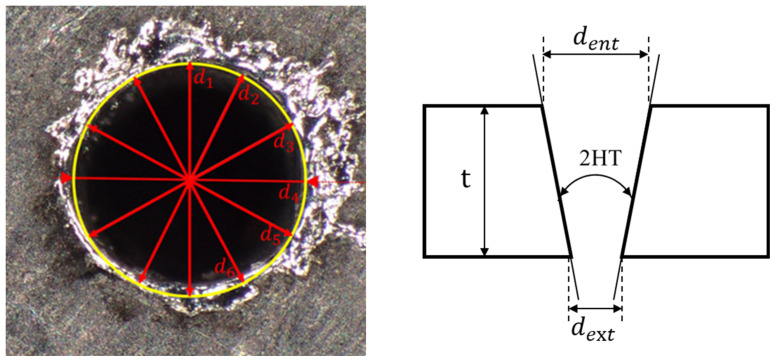
Schematic diagram of roundness and taper angle calculation.

**Figure A2:**
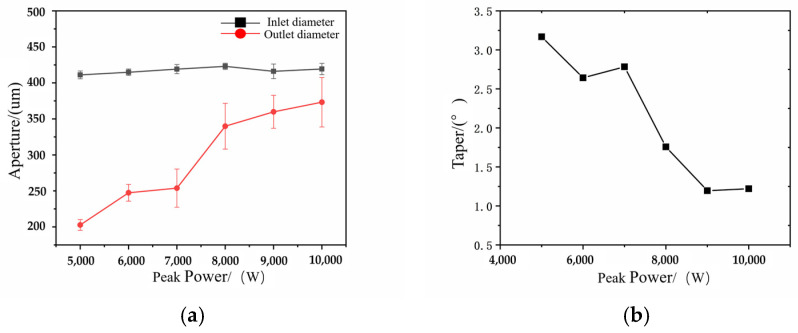
Diagram of aperture and taper variation with peak power. (**a**) Aperture variation diagram; (**b**) Cone variation diagram.

**Figure A3:**
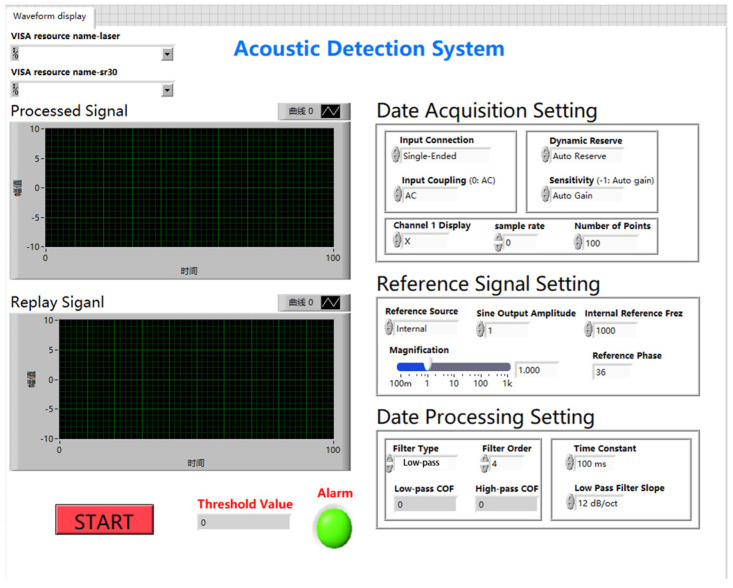
System parameter interface.

For the laser, set the peak power of the pulsed laser to 8000 W, with a duty cycle of 20%, repetition frequency of 1000 Hz, pulse width of 0.2 ms, laser wavelength of 1064 nm, defocal of 0 mm, focus spot diameter of 150 um and pulse interval of 100 ms. For the monitoring system, set the software to configure the phase—sensitive amplifier’s internal oscillator frequency to 35 kHz (The frequency response range of the acoustic sensor is 1–100 kHz), phase to 0°, signal input mode to differential input, filter slope to 24 dB/oct, sensitivity to 100 mV, data sampling rate to 1.667 kS/s, and system response time to 50 ms, which is less than the laser pulse interval of 100 ms. Use the method shown in the Figure 6 to conduct experiments and measure the taper and roundness of the hole. In the figure, “d_1_–d_6_” represent the diameters of the machined holes at different angles, “d_ent_” is the inlet diameter of the machined hole, ‘d_ext_“ is the outlet diameter of the machined hole, “t” is the thickness of the workpiece, and “2HT” represents twice the hole taper. The specific laser experiment date and acoustic system parameter setting interface are in the Table A1, Figure A2 and Figure A3. The data in Table A1 show that the mean roundness is 0.09766 and the variance is 0.000154 under different power levels. The mean taper angle is 1.99° with a variance of 0.68. This indicates that power variations have a greater impact on the taper angle.

### 3.2. Determination of Hole Penetration Threshold

**Figure 7:**
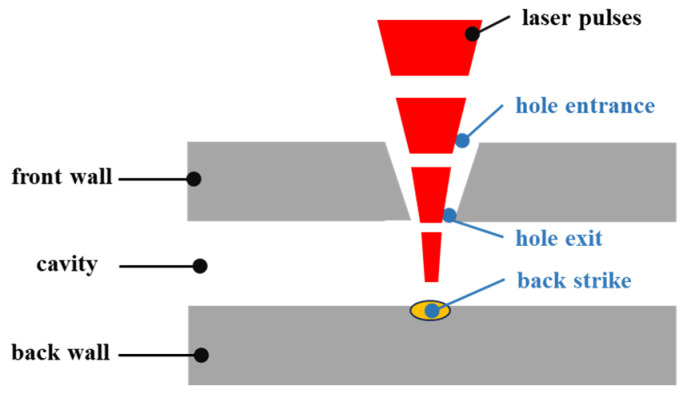
Laser processing diagram.

Based on the experimental parameters determined in Section 3.1, an acoustic monitoring experiment was conducted to investigate hole penetration. As illustrated in Figure 7, an additional plate of the same material was positioned 3 mm below the upper workpiece to simulate the backwall of a turbine blade. This setup allowed for the observation of the microscopic morphology of the backwall to assess potential damage caused by the laser drilling process. Under identical conditions, the drilling process was repeated five times using the same parameters to minimize experimental errors. The most representative result was selected for detailed analysis.

**Figure 8:**
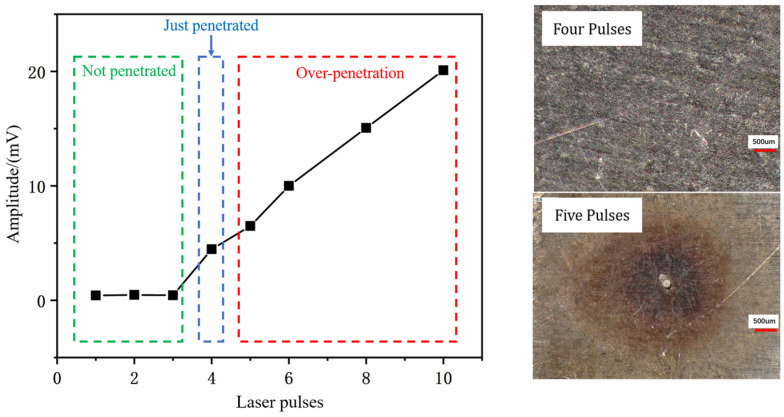
Relationship diagram between pulse quantity and signal strength and microscopic morphology of the backwall for four and five pulses.

Pulsed laser drilling was performed, and the corresponding acoustic signal amplitudes were recorded. Figure 8 shows the variation in signal amplitude as the number of pulses increased from 1 to 10. Concurrently, observations of the upper and lower workpieces revealed the following:

1. When the number of pulses was three or fewer, the upper workpiece remained unpenetrated.
2. At four pulses, the upper workpiece was fully penetrated, and no damage was observed on the backwall of the lower workpiece.
3. When the number of pulses exceeded four, the upper workpiece was penetrated, and varying degrees of damage were observed on the backwall of the lower workpiece.

Figure 7 also illustrates the microscopic morphology of the back wall for four and five pulses, providing a detailed comparison of the damage situation.

**Figure A4:**
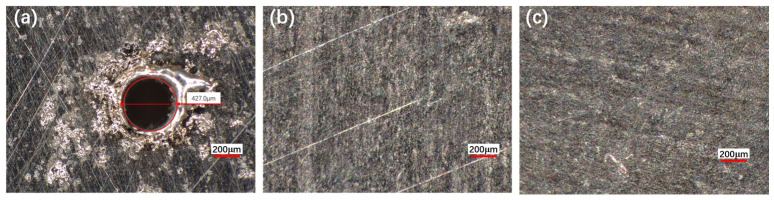
Apply a pulse to process the micro morphology of the entrance and exit of the hole and the back injury (**a**) Entrance of the hole; (**b**) Exit of the hole; (**c**)Back strike.

**Figure A5:**
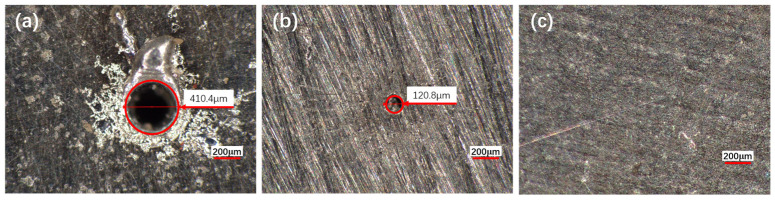
Microscopic morphology of the entrance and exit of the hole and the back injury during four pulse processing. (**a**) Entrance of the hole; (**b**) Exit of the hole; (**c**) Back strike.

**Figure A6:**
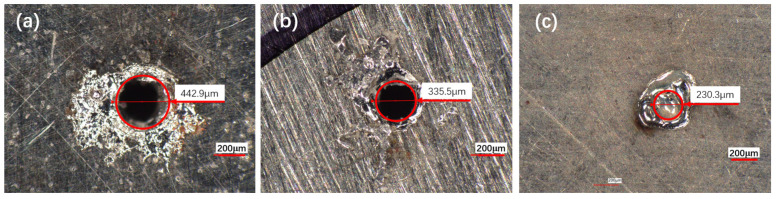
Microscopic morphology of hole entrance and exit and back injury processed by eight pulse processing. (**a**) Entrance of the hole; (**b**) Exit of the hole; (**c**) Back strike.

To better show the damage to the machined holes and opposite walls under different pulse counts, Figure A4, Figure A5 and Figure A6 presents the entrance/exit morphology of the machined holes and the damage to the opposite walls at 1, 4, and 8 pulses.

From the microscopic morphology of the backwall, it is evident that no damage occurs when the number of pulses is four. However, when the number of pulses increases to five, slight burning and bright spots appear on the backwall. Based on these observations, the signal amplitude corresponding to four laser pulses is identified as the hole penetration signal threshold under these parameter conditions.

**Figure 9:**
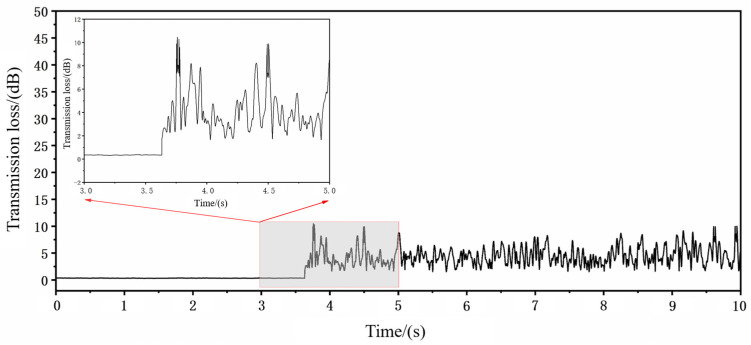
Signal variation chart of applying 4 pulses.

The variation in signal amplitude for four laser pulses, as shown in Figure 9, was analyzed in detail. Initially, the signal intensity is weak before drilling begins. When the laser starts drilling and penetrates the workpiece, the acoustic signal passes through the microhole and is captured by the microphone, causing the signal amplitude to surge above 2 mV and continue to rise. To further investigate the relationship between hole penetration and signal intensity, the signal intensity variation plot and statistical characteristics table were generated. As shown in the Table 2, the average signal intensity at this stage is 4.469 mV, which is more than ten times higher than the initial average signal intensity, indicating a significant change.

Based on these findings, 4.469 mV is selected as the hole penetration signal threshold for these parameter conditions, and subsequent experiments are conducted accordingly.

### 3.3. Investigation of the Relationship Between Signal Intensity and Hole Diameter

**Figure 10:**
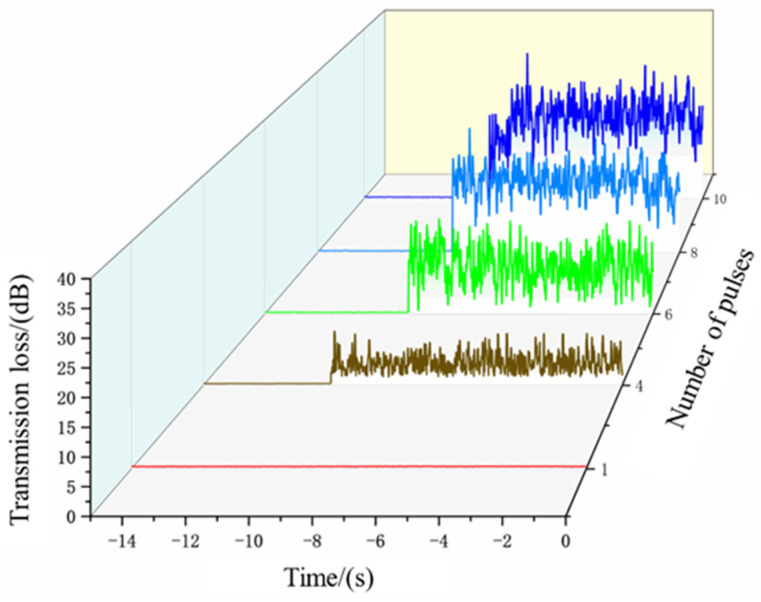
Relationship diagram between pulse quantity and signal strength.

To explore the relationship between the acquired signal intensity and the exit diameter of the drilled holes, the number of pulses was varied to produce holes with different exit diameters. Figure 10 illustrates the relationship between the number of pulses and the corresponding signal intensity.

To further investigate the relationship between signal intensity, exit diameter of the drilled holes, and the number of pulses, the post-breakthrough signals were extracted, and the corresponding exit diameters of the drilled holes were measured using a super-depth microscope. The resulting data are summarized in Table A2, which presents the statistical relationship between the number of pulses, exit diameter, and average signal intensity.

**Figure 11:**
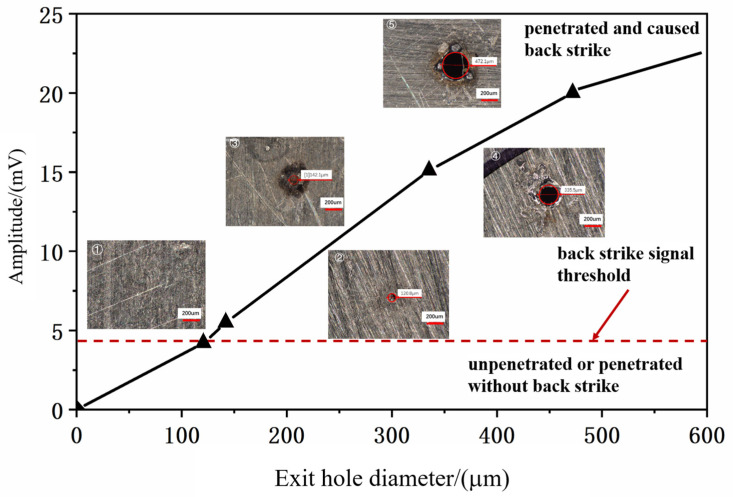
Hole exit diameter corresponding signal strength chart. (①~⑤ illustrate the aperture sizes under different signal strengths.).

To provide a more intuitive understanding of the relationship between the exit diameter of the drilled holes and the average signal intensity, Figure 11 illustrates the correlation between the exit diameter and the average signal value.

From the figure and table, it is evident that as the number of pulses increases, the exit diameter of the drilled holes gradually enlarges, and the average signal intensity also increases, demonstrating a clear linear relationship. Therefore, a linear relationship model was constructed to describe the correlation between the acoustic signal intensity (Y) and the hole diameter (X), yielding the following result:

(8) Y=0.0417X+0.413

This indicates that in subsequent experiments, the exit diameter of the drilled holes can be controlled or determined based on the signal intensity.

### 3.4. Investigation of Signal Variation Patterns During Multi-Hole Drilling

**Figure 12:**
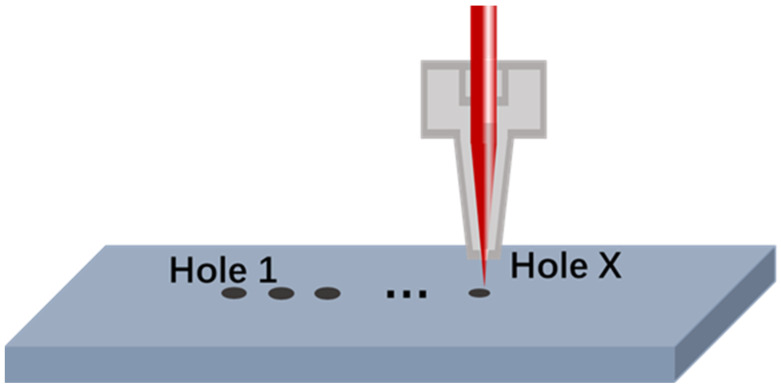
Multi-Hole drilling schematic diagram.

In practical laser drilling processes, it is often necessary to drill multiple holes. When using this laser drilling acoustic monitoring platform, previously drilled holes can influence the signals detected during subsequent drilling processes. Specifically, existing holes alter the propagation path of acoustic waves within the material. When laser drilling subsequent holes, the generated acoustic waves may reflect or scatter at the existing holes, leading to waveform distortion, amplitude attenuation, or time delays in the signals received by the sensor. To investigate the variation patterns of signal intensity during multi-hole drilling, a multi-hole drilling experiment was designed as illustrated in the Figure 12.

**Figure 13:**
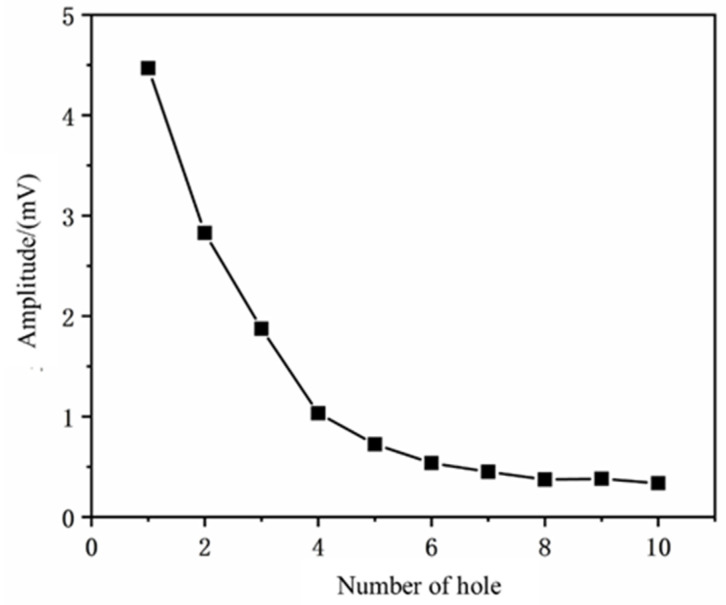
Relationship Between the Number of Drilled Holes and Average Signal Intensity.

Under the same laser and monitoring system parameters as described in Section 3.2, the microphone position was fixed, and the number of pulses is set to 4. Holes were drilled continuously on the workpiece at intervals of 2 mm. The average acoustic signal was recorded for each interval and plotted against the number of machined holes, resulting in the graph shown in Figure 13. The relationship between the specific signal intensity and the number of holes is shown in Table A3.

From the charts, it can be observed that during multi-hole drilling, the relationship between the number of holes and the average signal intensity follows an exponential decay trend. This is because the acoustic signals pass through the previously drilled holes and are received by the microphone, but the influence of these holes on the signal is significantly smaller than the impact of the distance between the drilling location and the microphone. To quantify the signal attenuation pattern, an exponential decay model was used to fit the relationship between the average signal intensity (I_L_) and the spacing (L). The fitting results are as follows:

(9) $$I_{L}=4.469mV\cdot e^{-0.31\cdot L}$$

Combining the results of the multi-hole drilling experiments, it is evident that multi-hole drilling does not affect the signal threshold for single-hole drilling. Specifically, when the microphone position remains fixed, the influence of previously drilled holes on the signal intensity is minimal. This is attributed to the use of high-frequency acoustic waves at 35 kHz, which have short wavelengths and concentrated energy, resulting in high directivity. The energy is primarily focused in the propagation direction, making it difficult for the acoustic waves to disperse and be received by the microphone. Therefore, during multi-hole drilling, previously drilled holes do not interfere with the penetration signal threshold for subsequent holes, making this approach advantageous for both single-hole or multi-hole drilling applications.

## 4. Discussion

This study proposes a hole penetration detection method for quasi-continuous laser drilling based on active acoustic signal monitoring. Utilizing the principles of the designed system, the monitoring system’s upper-level software was developed using LabVIEW graphical programming. The feasibility of the system’s acoustic monitoring module was verified using COMSOL acoustic simulation software, revealing the relationships between acoustic signal transmission loss, incident angle, and source frequency. The simulation results indicate that signal transmission loss decreases linearly with the source frequency. By comparing various datasets, the optimal source frequency was determined to be 35 kHz, with an optimal incident angle of 30°.

Using laser drilling parameters of 8 kW power, 1 kHz frequency, and 0.2 ms pulse width, the relationship between the number of laser pulses and signal intensity was established. Under these conditions, the hole penetration signal threshold without backwall damage was identified as 4.467 mV, corresponding to four laser pulses. Furthermore, based on experimental results, a proportional relationship between the drilled hole diameter and the average acoustic signal intensity was derived. The relationship between signal intensity (Y) and hole diameter (X) was found to satisfy Y = 0.0417X + 0.413, laying the foundation for controlling hole diameter through signal intensity in subsequent experiments. Finally, with the hole penetration threshold fixed at 4.467 mV and the microphone position unchanged, multi-hole drilling experiments were conducted. The results demonstrated that signal intensity decreases exponentially with increasing hole spacing, following the relationship I_L_ = 4.469 mV∙e^−0.31∙L^. This indicates that multi-hole drilling does not affect the signal threshold for individual holes, facilitating the determination of signal thresholds during single-hole or multi-hole drilling.

This study validates the feasibility of using acoustic principles for monitoring hole penetration states and measuring hole diameters, providing a theoretical and experimental foundation for preventing backwall damage during active laser drilling. By real-time monitoring of reference signal intensity to determine hole penetration states and providing instantaneous feedback to the laser control unit, the occurrence of backwall damage can be avoided. This ultimately achieves an active back-wall protection process for laser drilling in turbine blades. Furthermore, a linear relationship between signal intensity and machined-hole aperture was found, as well as a relationship between signal intensity and the number of machined holes in multi-hole machining. At present, this method has not yet been applied to actual processing. However, in the future, with further in-depth research on this method, including optimizing the system program or integrating it with AI technologies to enhance the system response speed and signal processing quality, it will be possible to achieve hole penetration detection and monitoring in the millisecond range. This method will not be limited by the processing material, will not be affected by environmental factors, and will be independent of the processing angle and position. This will significantly improve the efficiency and quality of gas film hole processing for aircraft blades. Moreover, this method can also meet the actual processing needs by using acoustic signal intensity detection or control of the hole diameter size of the processed hole.

## Figures and Tables

**Table 1:** Chemical composition of Inconel 718 superalloy.

Element	Cu	Mn	Co	Mo	Nb	Cr	Ni	Ti	Al
Mass Fraction/%	≤0.3	≤0.35	≤1	2.8~3.3	4.75~5.5	17.0~19.0	50.0~55.0	0.75~1.15	0.2~0.6

**Table 2:** Statistical table of signal characteristics.

Parameters	Numerical Values
Maximum value	10.45488886 mV
Median	4.208818585 mV
Minimum value	1.520186749 mV
Average value	4.468757777 mV
Initial average value	0.428234525 mV

## Data Availability

Data are contained within this article.

## Appendix A

**Table A1:** Relationship between aperture, taper and peak power.

Peak Power (W)	Inlet Diameter (μm)		Outlet Diameter (μm)		Roundness	Taper (°)
	Dmax	Dmin	Dmax	Dmin		
5000	415.56	405.17	208.45	194.32	0.974998	3.168315
6000	419.05	410.24	257.39	234.54	0.978976	2.642521
7000	424.69	412.27	282.61	230.31	0.970755	2.783823
8000	426.85	418.42	365.17	304.16	0.980251	1.757319
9000	426.69	406.66	385.91	343.24	0.953057	1.195296
10,000	426.52	411.06	409.65	341.29	0.963753	1.220800

**Table A2:** Pulse quantity-Processing hole outlet aperture-Average signal statistics table.

Number of Pulses	Aperture (μm)	Average Signal Strength (mV)
1	0	0.428
4	120.8	4.469
6	207.8	9.997
8	335.5	15.067
10	472.1	20.106

**Table A3:** Statistical table of multi-hole drilling.

Experiment Number	Number of Machined Holes	Average Signal Strength/(mV)
1	1	4.469
2	2	2.828
3	3	1.874
4	4	1.033
5	5	0.725
6	6	0.537
7	7	0.45
8	8	0.374
9	9	0.381
10	10	0.337
